# Mutational Biases and Selective Forces Shaping the Structure of Arabidopsis Genes

**DOI:** 10.1371/journal.pone.0006356

**Published:** 2009-07-27

**Authors:** Salvatore Camiolo, Domenico Rau, Andrea Porceddu

**Affiliations:** Dipartimento di Scienze Agronomiche e Genetica Vegetale Agraria (DISAGEVA), Università degli Studi di Sassari, Sassari, Italy; University of Umeå, Sweden

## Abstract

Recently features of gene expression profiles have been associated with structural parameters of gene sequences in organisms representing a diverse set of *taxa*. The emerging picture indicates that natural selection, mediated by gene expression profiles, has a significant role in determining genic structures. However the current situation is less clear in plants as the available data indicates that the effect of natural selection mediated by gene expression is very weak. Moreover, the direction of the patterns in plants appears to contradict those observed in animal genomes. In the present work we analized expression data for >18000 *Arabidopsis* genes retrieved from public datasets obtained with different technologies (MPSS and high density chip arrays) and compared them with gene parameters. Our results show that the impact of natural selection mediated by expression on genes sequences is significant and distinguishable from the effects of regional mutational biases. In addition, we provide evidence that the level and the breadth of gene expression are related in opposite ways to many structural parameters of gene sequences. Higher levels of expression abundance are associated with smaller transcripts, consistent with the need to reduce costs of both transcription and translation. Expression breadth, however, shows a contrasting pattern, i.e. longer genes have higher breadth of expression, possibly to ensure those structural features associated with gene plasticity. Based on these results, we propose that the specific balance between these two selective forces play a significant role in shaping the structure of *Arabidopsis* genes.

## Introduction

Several studies conducted in organisms as diverse as humans, *Drosophila melanogaster* and *Caenorhabidtis elegans*, have demonstrated that there is an inverse relation between levels of gene expression and a variety of sequence parameters such as the length of coding sequence, intron number and length [1]–[3]. These patterns have inspired the energetic cost hypothesis under which natural selection would favour shorter transcriptional units to minimize time and cost of gene expression [1]. However, alternative interpretations have not been ruled out. For example, the *genomic by design* model postulates that the activity of a gene is a key determinant of its structure. According to this hypothesis, genes that work in only a few tissues and thus require a high level of epigenetic regulation, have a structure particularly rich for non coding sequences to host the necessary regulatory elements [3], [4]. The observation that tissue specific human genes have more introns and longer intergenic sequences than broadly expressed ones lends credit to this proposition [4], [5]. A third hypothesis points toward a local mutational bias as the main force controlling for gene structure. For example, Urrutia and Hurst [2] have suggested that broadly expressed human genes are positioned in genomic regions more prone to deletions and for this reason are shorter than tissue specific ones. The mutational bias could be even more focused on gene sequences. Highly abundant transcripts would be more prone to reverse transcription and retroposition and this would explain the lower density of intronic sequences in highly transcribed regions [6].

This debate has recently experienced a puzzling turn due to the contradictory results presented for plant genomes. Seoighe et al. have shown that genes expressed in the *Arabidopsis* male gametophyte have shorter introns than genes that are expressed exclusively in the sporophyte. This observation provided the first evidence of a molecular signature of strong gametophytic selection and was considered in agreement with the energetic cost hypothesis [7]. Yet, subsequent studies in *Arabidopsis* and rice genomes have shown that the relationships between gene expression level and length in plants depict just the opposite trend: highly expressed genes are the least compact having more and longer introns than lowly expressed ones [8], [9]. Given these contradictory results, it is not possible to establish to what extent selective pressures in plants differ from previously studied metazoan genomes. To address this issue we compared estimates of gene expression against several gene characteristics. For this purpose we assembled expression data from publicly available oligo-array [10] and MPSS (Massively Parallel Signature Sequencing) experiments [11]. Our results provide evidence that expression level and breadth are related to gene sequence characteristics in opposite ways. Higher levels of gene expression are associated with smaller transcripts whereas greater breadth of expression correlates with longer transcripts. The balance between these two selective forces represents a significant determinant of the structure of *Arabidopsis* genes.

## Methods

### Sequence information

Sequence information for protein coding genes was obtained from TAIR 8 annotations (ftp://ftp.arabidopsis.org/home/tair/Sequences/). Transposons, pseudogenes, plastid and mitochondrial genes were filtered out from the data set. Although 23528 fully annotated transcript entries were available for the analysis, data for each structural parameter were not obtained for all the genes and therefore the actual number of genes used in comparisons varied as indicated in the text. In all cases in which more than one alternative transcript was predicted, the longest was analysed.

### Expression data

We used gene expression data from two web sources. Oligo array data were retrieved from the http://www.ncbi.nlm.nih.gov/sites/entrez (see supplemental table S1 for a complete list of experiments considered) [10]. They represent the results of oligonucleotide array experiments performed uniformly with a total of 77 developmental stages belonging to different organs [10]. The signals from probes on the chip corresponding to the same gene were normalized using the RMAexpress software [12] (also the replicates representing the same developmental stages were averaged. A gene was regarded as expressed if its signal level exceeded a conservative threshold of 75 average difference value.

MPSS data were retrieved from http://mpss.udel.edu/at/ (See for a complete list of the experiments considered) and only tags matching a single gene were considered as described by Meyers et al. [11]. The five libraries analysed represented five organs: leaves, roots, germinating seedlings, flowers and siliques.

### Measures of gene expression

Gene expression profiles were measured in two ways: i) breadth and ii) level of expression. The *breadth of expression* of a gene (EB) takes into account the number of experimental units (organs or developmental stages) in which a gene is expressed. The level of expression of a gene provides a quantitative estimation of mRNA accumulation in a experimental unit and was obtained in several ways: the peak of expression (pE) which represents the highest value of expression of a gene across all experimental units, and the mean level of expression calculated taking into account all experimental units (hereafter referred to as A) or, only those in which the expression is not zero (hereafter designed as informative experimental units I). In addition, two possible definitions of experimental units were used. In the (O) organ based definition the data from developmental series were pooled together, while in the (DS) definition each developmental stage of an organ represented a unique experimental unit.

By combining expression variables and experimental unit definitions six different methods for measuring gene expression were established. For a complete list of measures of gene expression see table S3 in supplemental material.

#### DS methods

Each developmental stage represented an experimental unit and expression measures for each gene were averaged either considering all experimental units (DS-A) or only informative ones (DS-I):. DS-pE represents the peak of expression identified taking into account all DS experimental units.

#### O methods

Each organ represented an experimental unit. Expression measures for a given gene were calculated by averaging the values of either all organs (O-A) or only those with expression different from zero (O-I). O-pE is the peak of expression: i.e the highest value among O-defined experimental units

Expression breadth was calculated considering DS experimental units (DS-EB) or organ defined experimental unit (O-EB).

### Statistical analyses

Indexes of expression levels and genic parameters were log transformed prior to analysis. Pearson' s (parametric) correlations (r) were calculated on single genes data and graphs were drawn binning by expression (bin = 5% of the dataset). Multiple regression analyses were performed on standardized variables.

## Results

If selection is acting on gene sequences to maximize expression efficiency, we might expect to find a relationship between gene expression profiles and some descriptions of sequence parameters. Expression profiles were reduced to discrete measures considering all (A-methods) or only informative (I-methods) experimental units which were based on single observations (DS) or organ series (O). Three descriptive variables of gene expression profiles were considered: the average level of expression (EL), the peak of expression (pE) and the breadth of expression (EB).

To study information redundancy we applied cluster analysis on multivariate correlations between estimates obtained with different methods. It is worth to underline here that, in principle, the two averaging methods (A versus I) are expected to have a different propensity for representing quantitative differences between expression profiles. In facts, because the A averaging methods minimizes the weight of single data, A-based measures are expected to be more dependent on expression breadth [2].

**Figure 1:**
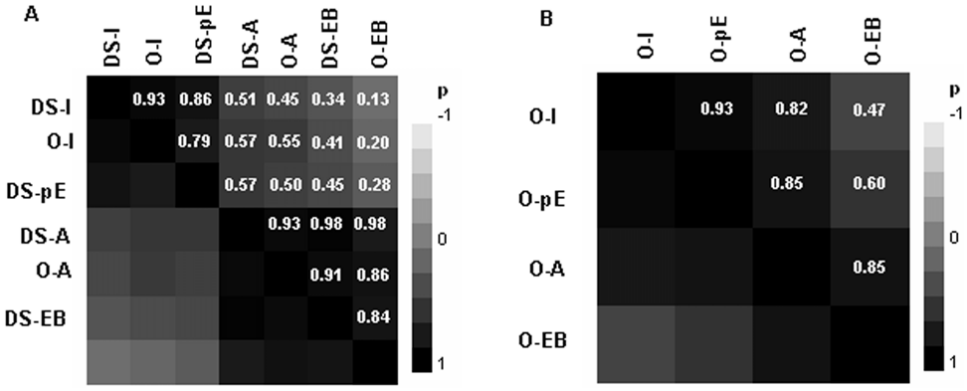
Cluster on the correlations (Pearson's *r*) among the different measures of expression considered both for (A) oligo-array and (B) MPSS. The experimental unit is represented by the developemental stage in the DS method and by the Organ in the O method. Expression profiles were reduced to discrete measures considering all (A-methods) or only informative (I-methods) experimental units.

As a matter of fact, for oligo-array (Figure 1-a), the way of collecting data (A vs I) discriminated between EL methods more than the definition of experimental units (DS vs O). As expected, we found that expression breadth measures were closer to EL measures with A definition, whereas the pE measure was positioned between I and A-EL (Figure 1-a). The results from MPSS (Figure 1-b) were similar, though of a lower resolution.

To represent the whole range of variation we considered here the most independent measures: DS-I and DS-EB for oligo-array and O-I and O-EB methods for MPSS. For complete information on all six methods the reader is addressed to supplemental tables (table S3).

### The length of genic and intergenic regions

Time and costs of transcription are proportional to the length and amount of the transcript that is produced. Thus, according to the selection for energy cost hypothesis, highly expressed genes are likely to experience greater selective pressure for a reduction in transcript length [1].

**Figure 2:**
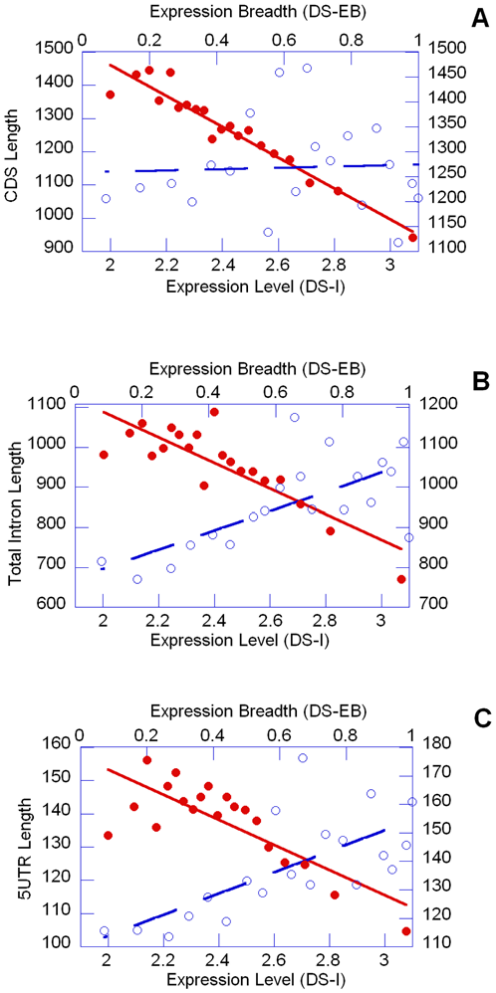
Relationship between the expression profile and (a) the CDS length, (b) the Total Intron Length and (c) 5utr Length. The points indicated by the symbol • and interpolated by the continuous line refer to the expression level, whereas the points indicated by the symbol ○ and interpolated by a dashed line refer to the expression breadth. In both cases 14236 genes were considered which have been grouped in bins, each representing the 5% of the whole dataset.

We found that indeed expression levels and primary transcript length were negatively correlated (e.g. oligo-array DS-I: r = −0.142 n = 14236 P<0.0001) and this was common to almost every transcript component indicating the presence of a generalized effect. In fact, total intron, 5′ UTR and, noticeably, also CDS lengths were negatively correlated to expression level (Table 1 and Figure 2). A significant tendency towards reduction was found also for the total number of introns per gene (e.g. oligo-array DS-I r = −0.079 n = 14236 P<0.0001; for details see Table 2). The only exception to such a scenario was the positive correlation between 3′ UTR and expression level (e.g. oligo-array DS-I r = 0.074 n = 14236 P<0.0001).

**Table 1:** Correlation between structural genomic parameters and both gene expression and expression.

Gene characteristics	*Expression level*		*Expression breadth*	
	*Microarray*	*MPSS*	*Microarray*	*MPSS*
	DS-I	O-I	DS-EB_w0	O-EB_w0
	*r*	*r*	*r*	*r*
Gene length	−0.142*	−0.064*	0.136*	0.108*
5′ UTR length	−0.023†	0.030‡	0.154*	0.125*
CDS length	−0.164*	−0.125*	0.014	0.014 ns
Intron length	−0.022†	0.018§	0.174*	0.124*
Intron length_w0	−0.102*	0.014 ns	0.152*	0.141*
3′ UTR length	0.074*	0.141*	0.202*	0.178*
Number of exons	−0.079*	−0.015	0.132*	0.099*
Average exon length	−0.028*	−0.061*	−0.159	−0.084*
Number of introns	−0.079*	−0.015*	0.132*	0.099*
Number introns_w0	−0.099*	−0.016 ns	0.090*	0.099*
Average intron length	0.008 ns	0.045*	0.146*	0.042*
Average intron length_w0	0.003 ns	0.050*	0.060*	0.044*
Intron Density	0.007 ns	0.078*	0.188*	0.124*
Intron Density_w0	0.004 ns	0.078*	0.151*	0.124*

Data are presented both for oligo-array and MPSS assays. Statistical significances: ns = not significant; §P<0.05 †P<0.01; ‡P<0.001;*P<0.0001. Sample size of the correlations were comprised between n = 12051 and n = 14236. **Note1.** For this calculations, gene for which expression was zero were not considered to avoid potential problem arising from defective probes (Eisenberg and Levanon, 2003). **Note2.** For introns parameters, data are presented both considering and excluding (w0) genes without introns.

**Table 2:** Correlation between structural genomic parameters and both gene expression level and expression breadth.

Genomic variables	*Expression level*		*Expression breadth*	
	*Microarray*	*MPSS*	*Microarray*	*MPSS*
	DS-I	O-I	DS-EB_w0	O-EB_w0
GC% Gene	0.033*	0.043*	0.046*	0.084*
GC% 5′ UTR	−0.077*	0.021§	0.156*	0.137*
GC% CDS	0.222*	0.254*	0.109*	0.084*
GC% intron_w0	−0.079*	0.025†	0.286*	0.234*
GC% 3′ UTR	−0.045*	0.000 ns	0.124*	0.124*
Length of intergenic spacers	0.027†	0.017†	−0.109*	−0.063*
Length of intergenic spacers_w0	0.024†	0.016 ns	−0.123*	−0.069*
GC% intergenic spacers	0.008 ns	0.000 ns	0.076*	0.045*
GC% of RNA	0.074*	0.115*	0.121*	0.093*

Data are presented both for oligo-array and MPSS assays. Statistical significances: ns = not significant; §P<0.05 †P<0.01; ‡P<0.001;*P<0.0001. Sample size of the correlations were comprised between n = 12051 and n = 14236. **Note.** For this calculations, gene for which expression was zero were not considered to avoid potential problem arising from defective probe (Eisenberg and Levanon, 2003).

The picture emerging from MPSS data was less clear as expected given the lower resolution of the data. Both CDS and total intron length followed the pattern toward reduction (see Table 1). Intron number was not significantly correlated with expression level and 5′UTR, joined 3′UTR in marking the opposite trend toward expansion (Table 1).

In addition we studied a measure of intron density defined as the number of introns per kb of CDS [13]. Expression level was positively correlated to intron density for MPSS experiments and not correlated for oligo-array experiments (Table 1). This result is not caused by low expression of intron less genes because a similar trend was observed also when only intron containing genes were considered in the analysis.

EB measures for both oligo-array and MPSS were positively correlated to transcript length and this tendency was conserved also for 5′ UTR, 3′ UTR and total intron length and number. Conversely the CDS was not correlated to EB either in oligo-array or in MPSS data. As expected intron density was positively correlated to expression breadth for both oligo-array and MPSS experiments (Table 1).

Finally, we studied the relationships between expression profiles and the length of intergenic sequences. Noteworthy expression breadth, which is associated with gene sequence expansion, was negatively correlated with the length of intergenic sequence (e.g for oligoarray DS-EB r = −0.109 n = 14526 P<0.0001; see Table 2).Expression level, on the contrary was weakly, positively, correlated to the lengths of intergenic spacers (e.g. for DS-I r = 0.027 n = 14236 P<0.01).

### GC content

GC content of genes can account for several DNA physical features potentially associated to gene expression. For example, the bendability of DNA increases faster with the elevation of GC content and curvature drops faster than in random sequence ([14], [15]. The former property is considered to be associated with open chromatin usually linked to active transcription while the latter with condensed chromatin associated to repressed transcription states ([16], [17], [18]. Also, the thermostability of RNA/DNA and RNA/RNA complexes increases faster with the elevation of GC content which suggests implications in transcription regulation or sense/antisense transcript interactions [14].

In Arabidopsis genome the GC content of coding sequence was positively correlated to both expression level and breadth (Table 2). Non coding gene sequences, showed different patterns between expression breadth and level: introns and UTRs were positively correlated to expression breadth and negatively or not even associated to expression level. Also the GC content of intergenic spacers followed a similar pattern, being positively correlated to expression breadth and not associated to expression level.

### Direct effects and regional mutation biases

Because the measures of expression level and expression breadth are highly correlated with each other (see Figure 2) we reconsidered the effects of either of the two measures on gene sequences after correcting for the other. In general, the results of the multiple regressions shown in table 3 confirmed and strengthen the patterns described by the pairwise correlations: i.e the level of expression being negatively correlated to gene parameters and breadth of expression depicting the opposite trend in favour of expansion. The only noticeable exception was the absence of a significant relationship of expression level, calculated by oligo array data, with 3′UTR after correcting for expression breadth.

**Table 3:** Multiple regression analysis of EL, EB and several gene parameters.

	Microarray						MPSS					
	Independent variables in the models						Independent variables in the models					
Dependent variable	EL,EB		EL,EB & regional^1^		EL,EB, regional & genic^2^		EL,EB		EL,EB & regional^1^		EL,EB, regional& genic	
	β_EL_	β_EB_	β_EL_	β_EB_	β_EL_	β_EB_	β_EL_	β_EB_	β_EL_	β_EB_	β_EL_	β_EB_
5′ length	−0.084*	0.181*	−0.089*	0.195*	−0.046*	0.108*	**−0.035‡**	0.135*	−0.041*	0.143*	−0.021§	0.085*
CDS length	−0.176*	0.073*	−0.179*	0.078*	−0.095*	−0.025†	−0.155*	0.086*	−0.160*	0.094*	−0.118*	**0.026†**
Intron length^3^	−0.175*	0.214*	−0.177*	0.219*	−0.065*	0.095*	−0.066*	0.169*	−0.066*	0.169*	−0.010^ns^	0.047*
3′ length	0.005^ns^	0.188*	0.001^ns^	0.197*	0.035‡	0.128*	0.065*	0.127*	0.063*	0.131*	0.076*	0.080*
PT length	−0.205*	0.200*	−0.206*	0.204*	n.a.	n.a.	−0.125*	0.150*	−0.129*	0.157*	n.a.	n.a.
Intron number^4^	−0.150*	0.144*	−0.148*	0.140*	0.027*	−0.029*	−0.080*	0.138*	−0.080*	0.136*	0.020†	−0.010^ns^

Results from multiple-regression analyses of level of gene expression (EL) and breadth (EB) and length (of 5′, CDS, intron, 3′ and primary transcript, PT) when controlling for regional and genic effects. The results for the intron number are also shown. All lengths were log_10_ transformed.

**Note.** PT = Primary Transcript; n.a. = not applicable; Significance levels: ns = not significant; §P<0.05 †P<0.01; ‡P<0.001;*P<0.0001. **In bold:** values that are significant after Bonferroni correction for multiple regression. Alpha level was adjusted separately for each type of model: EL,EB: 0.05/2 = 0.025; EL,EB & regional: 0.05/4 = 0.0125; EL,EB, regional+genic = 0.05/9 = 0.0056 (see Mundfrom et al 2006).

1 regional variables: intergenic spacers length and intergenic spacers GC content. Genes without intergenic spacers were excluded.

2 genic variables; 5′,CDS,Intron,3′, primary transcript lengths and intron number. Genes without introns were not included in the analyses.

3 ,^4^genes without introns were excluded from the analyses.

Recently, Urrutia and Hurst have shown that regional mutational biases may influence the local level of insertions and deletion in the human genome [2]. Also compositional issues have been related to structural features. For example, based on GC contents of gene sequence, Carels and Bernardi have identified two classes of genes which exhibit distinctive structural features [19].

In the previous section we have described the relationships between measures of gene expression and the length and the GC content of intergenic sequences. To verify the possibility that these correlations, could explain the relationship between expression profiles and gene characteristics we studied the consequences of including the length and GC content of intergenic sequences as additional independent variables in our models. As shown in table 3, the correction for regional variables showed only a limited impact on the effects of expression profiles on gene characteristics (Table 3).

### Intra-genic effects

Since the lengths of genic regions are known to be highly correlated each other, we investigated the nature of the dependencies between expression profiles and length of genic regions. Each relationship between expression and length of genic regions was, thus, reconsidered after correcting for the effects of other regions of the gene and regional variables. The corrections had a strong impact on some of the relationships and in some cases the beta coefficients were no longer significant after the Bonferroni correction (see Table 3). All together, these results confirmed the scenarios previously described. Expression level was negatively correlated to gene sequences, with the only exception of 3′ UTR. The results for expression breadth confirmed its positive relation with both UTRs and introns whereas the correlations for CDS again differed between MPSS (positively correlated) and oligo-array (negatively correlated).

Notably, the largest corrections were noticed for CDS and total intron lengths, while the least varied coefficients were found for the 3′ UTR.

To gather a deeper insight on the dynamics of intron length and number variations we analysed the effect of expression profile with respect to intron position. Several studies, that have combined data from multiple species, have found that the first intron is,on average, longer that other introns [20]. A proposed explanation for this trend is that introns from the 5′ proximal region of a gene have important functional features related to gene expression while introns from the middle or 3′ end of genes have progressively lower impacts. Recently, Rose et al. have demonstrated that signals responsible for boosting the expression are most abundant in introns near the transcription start site and that the compositional difference between promoter proximal introns and distal introns can be used to predict the ability of an intron to stimulate expression [21]. In a subsequent paper, Bradman and Korf [20]. have demonstrated that density of enhancing signals decreases with intron order. In the effort to minimize the cost of gene transcription, the action of selection should preferentially be directed toward distal introns. According to this hypothesis the strength of the miniaturization effect on intron length should steadily increase with intron order.

Multiple regression analyses were performed considering intron length as dependent variable and EL and EB as independent variables. For each order of intron position, β coefficients were collected both for EL (β_EL_) and EB (β_EB_).

**Figure 3:**
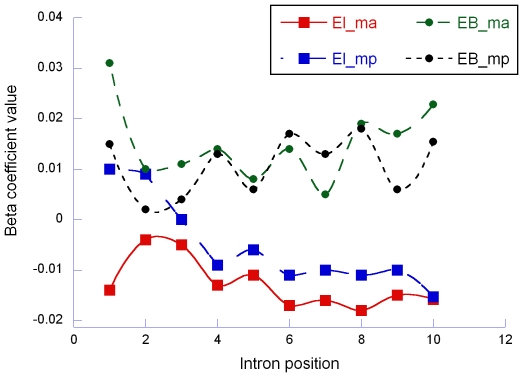
Results of multiple regression analyses between intron length and espression level (EL) and breadth (EB) by intron position. The analysis was conducted considering both microarray (ma) and MPSS (mp) data and all genes. Full circles: significant at P<0.05; empty circles: not significant at P>0.05.

The β_EL_ coefficients depended by intron order (figure 3): proceeding from 5′ to 3′ ends, the β_EL_ values became more negative remaining significant in spite of the reduction of sample size. For oligo-array, 42.1% (Spearman ρ = −0.699, n = 10, P = 0.0245) of the variance of β_EL_ coefficients was explained by intron position and for MPSS this proportion reached the 78.6% (ρ = −0.924, n = 10, P = 0.0001). No significant trends (Spearman P>0.16) were observed for β_EB_ (only 13.1% and 10.0% of the variance explained by intron position for microarray and MPSS, respectively). Interestingly, after removing the first intron, negative trends were confirmed for β_EL_ and positive trends were indicated for β_EB_. Indeed, the proportion of explained variance become 63.3% (ρ = −0.753, P = 0.0190; for microarray) and 70.6% (ρ = −0.895, P = 0.0011; for MPSS) for β_EL_ and 34.0% (ρ = 0.630, P = 0.0688; for microarray) and 40.1% (ρ = 0.679, P = 0.0442, for MPSS) for β_EB_.

**Figure 4:**
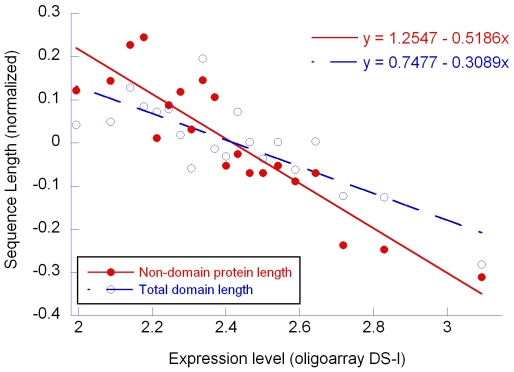
Relation between the expression level (oligoarray DS-I) and the Total Domain Length and Non-domain protein length. Variables on the Y axis have been normalized

In the previous section we have demonstrated that natural selection mediated by expression level exerts its action preferentially on distal introns owing to their lower importance for gene expression. With an analogous construction we may hypothesize that natural selection acts on CDS reducing, preferentially, the sequences coding for amino acids not strictly essential for protein function. Indeed we found that within CDSs the slope of the linear regression between the length of the regions outside functional domains and expression level was about 1,7 fold higher than the slope of the linear regression between the length of functional protein domain and expression level (Figure 4). As expected the EB behaved differently with the length of CDS coding for functional domains increasing while the remaining part decreased.

## Discussion

The results presented here suggest that in *Arabidopsis* genome, structural parameters of genes are related to expression level and expression breadth in opposite directions. The level of expression is assocated with a generalized tendency toward transcript miniaturization while the breadth, i.e the number of experimental units in which a gene is expressed, is associated with sequence expansion.

Are these patterns consistent with the action of natural selection, or can they be explained by neutral processes in the Arabidopsis genome?

To answer this question we resorted to a multiple regression analysis of regional genomic characters that could potentially explain the relationships between expression profiles and gene structure. The considered regional variables only partially accounted for the relationships between the level and breadth of gene expression and gene sequences. These findings were not conclusive in favor of selection, as it can be argued that other regional variables, not explored in our analysis, might account for the remaining parts of the effects.

Instead of embarking in a cumbersome search of these additional regional variables, we adopted a more qualitative approach to identify signatures specific of selection. We began by proposing that selective process acting on genic sequences to maximize expression efficiency should discriminate between sequence dispensability. In contrast, regional mutational biases acting alone should have more generalized effects. Following such an approach, we found two evidence of signatures of selection. First, the miniaturization effect of selection in highly expressed genes is stronger in the more distal introns. This evidence is in line with a recent finding in plants that indicates that the signals responsible for boosting expression are more abundant in proximal than in distal introns [21]. Second, the action of selection in highly expressed genes is preferentially directed toward sequences outside the regions coding for functional domains. Taken together these observations suggest that natural selection is directly acting on gene sequences to maximize the efficiency of expression and that regional variables may, eventually, concur to this action.

Are these effects similar to those observed in other non plant genomes, or do they represent unique features of plant genomes? Before trying to answer this question, it is important to remember, that the level of resolution of our analysis was achieved considering the least correlated definitions of expression level and breadth. Other definitions of expression level, such as those weighing the experimental units with no expression, produced less defined picture [8], [9].

While the miniaturization effect is in line with the selection for economy model, the effect observed on genes with high EB, in *Arabidopsis*, is in apparent striking contrast with observations in other systems. At least three scenarios may have generated this dichotomy: i) the forces shaping plant genes are the same as those affecting other genomes but subject to a different balance ii) the nature of selective forces are unique to plant genomes iii) a mix of the first two scenarios.

Let's analyse the different options moving from the homologies between different genomes. Average intron density is correlated to expression breadth in both humans [13] and *Arabidopsis*. Comeron, has speculated that this association could be related to the influence of introns on mRNA metabolism and splicing efficiency [13]. During splicing, a complex of several proteins called exon-exon junction complexes (EJC) are deposited on processed mRNA in proximity of splicing sites [22]. Recent evidence has indicated that this complex would exert a post transcriptional enhancing effect, influencing export efficiency of mRNA to the cytosol and promoting transcriptional elongation and translation. Thus, according to this view, the number and total length of introns per gene results from two opposing selective forces: one favoring their proliferation because of the beneficial effects of EJC on some aspect of general RNA metabolism, and the other working for their reduction because of the cost associated to their transcriptions. An additional force favoring intron number and length increase could be connected to gene plasticity. Kreitman and Wayne have proposed a selective model for intron presence associated with the deleterious effects of linkages between sites under selection, a phenomenon termed Hill-Robertson effect. Indeed, linkage between selected loci can reduce the overall effectiveness of selection and the rate of adaptation [23]. This phenomenon also generates indirect selection to increase recombination rates. Introns generally have a reduced frequency of sites under selection compared to exons. Thus, an increase in intron number and length will increase the distance between mutations under the influence of selection in adjacent exons. This will favor recombination events between selected sites on different exons and consequently would improve the responsiveness of gene to selection.

Number and length of introns could also be related to the expression breadth in term of balancing selection. Wegmann et al. have recently demonstrated that human genes producing politype transcripts are expressed in a larger number of tissues and have more introns than genes producing monotype transcript [24]. The authors suggested that genes with high expression breadth and high intron number will be more prone to produce new transcript isoforms which could be maintained because ensuring an higher adaptability to various tissues conditions. Following this reasoning we analyzed alternatively spliced Arabidopsis genes and found that they show the tendency to have a wide expression breadth and longer primary transcript with more and longer introns (data not shown). Therefore, in this respect Arabidopsis genes known to produce polytype transcripts are similar to human alternatively spliced genes.

Under the first scenario, different balances between selective forces would account for the differences in selective signatures. For example, it is possible that selection for miniaturization in human genes would overcome the effects of the other forces. A very important genic parameter to consider in this context is the overall gene structure. For example in the human genome the average intron length is 5.5 kb, which is considerably larger than 152 bp of the average intron length of *Arabidopsis* genes [8]. Human genes have on the average also more introns than *Arabidopsis*. Broadly expressed human genes would be more compact than narrowly expressed ones in consequence of the high correlation between expression level and breadth. The observation that the difference in compactness between broadly expressed and narrowly expressed human genes loses significance when the comparison between these two categories is carried out taking into account the level of expression, lends support to this hypothesis [25]. In *Arabidopsis* genome, on the contrary, the marginal gain in fitness due to intron reduction and miniaturization in broadly expressed genes could be lower, due to the shorter average length and lower average number of introns per gene. Thus in spite of its high level of correlation with expression level, the breadth of expression shows, in the *Arabidopsis* genome, a prevalent effect toward sequence expansion.

Alternative interpretations may be hypothesized calling into the field plant specific selective forces (scenario 2). For example, compositional differences between plant and human introns may have have different consequences for gene expression regulation. The nucleosome binding sites may have a different distribution between introns and exons in plant genes compared to human genes and this may be a specificity of plant introns favouring the establishment of open chromatin configurations which are typical of genes with high EB.

Further investigations in other plant species having different genome size, life cycle, and reproductive system (for example vegetative vs sexual reproduction) will add elements to either of the two scenarios analyzed or configure new pictures by combining new and old elements (our scenario three).

## Supporting Information

Table S1 List of oligoarray experiments considered in our paper
(0.17 MB DOC)

Table S2 List of MPSS experiments considered in our paper
(0.03 MB DOC)

Table S3 Methods used in order to estimate the gene expression levels.
(0.03 MB DOC)
