# Supplementary material for: Mutational Biases and Selective Forces Shaping the Structure of Arabidopsis Genes
Source: PLoS One. 2009 Jul 27;4(7):e6356. doi: 10.1371/journal.pone.0006356 (PMC2712092; doi:10.1371/journal.pone.0006356)
Supplement: Table S1 — List of oligoarray experiments considered in our paper (0.17 MB DOC) [file pone.0006356.s001.doc]

**Table S1:** List of all oligoarray experiments considered

| **Series:**  Seedlings and whole plants | | | | | | |
| --- | --- | --- | --- | --- | --- | --- |
| **Available at:**  http://affy.arabidopsis.info/narrays/experimentpage.pl?experimentid=149 | | | | | | |
|  |  |  |  |  |  |  |
| **GEO Access Identifier** | **Experiment Name** | **Tissue** |  |  |  |  |
|  |  |  |  |  |  |  |
| GSM131471 GSM131472 GSM131473 | ATGE_7_A2 ATGE_7_B2 ATGE_7_C2 | seedling, green parts |  |  |  |  |
|  |  |  |  |  |  |  |
| GSM131474 GSM131475 GSM131476 | ATGE_22_A ATGE_22_B ATGE_22_C | whole plant after transition |  |  |  |  |
|  |  |  |  |  |  |  |
| GSM131477 GSM131478 GSM131479 | ATGE_23_A ATGE_23_B ATGE_23_C | whole plant after transition |  |  |  |  |
|  |  |  |  |  |  |  |
| GSM131480 GSM131481 GSM131482 | ATGE_24_A ATGE_24_B ATGE_24_C | whole plant after transition |  |  |  |  |
|  |  |  |  |  |  |  |
| GSM131483 GSM131484 GSM131485 | ATGE_96_A ATGE_96_B ATGE_96_C | seedling, green parts |  |  |  |  |
|  |  |  |  |  |  |  |
| GSM131486 GSM131487 GSM131488 | ATGE_97_A ATGE_97_B ATGE_97_C | seedling, green parts |  |  |  |  |
|  |  |  |  |  |  |  |
| GSM131489 GSM131490 GSM131491 | ATGE_100_A ATGE_100_B ATGE_100_C | seedling, green parts |  |  |  |  |

| **Series:**  Roots | | | | | | |
| --- | --- | --- | --- | --- | --- | --- |
| **Available at:**  http://affy.arabidopsis.info/narrays/experimentpage.pl?experimentid=151 | | | | | | |
|  |  |  |  |  |  |  |
| **GEO Access Identifier** | **Experiment Name** | **Tissue** |  |  |  |  |
|  |  |  |  |  |  |  |
| GSM131555 GSM131556 GSM131557 | ATGE_3_A ATGE_3_B ATGE_3_C | root |  |  |  |  |
|  |  |  |  |  |  |  |
| GSM131558 GSM131559 GSM131560 | ATGE_9_A ATGE_9_B ATGE_9_C | root |  |  |  |  |
|  |  |  |  |  |  |  |
| GSM131561 GSM131562 GSM131563 | ATGE_93_A ATGE_93_B ATGE_93_C | root |  |  |  |  |
|  |  |  |  |  |  |  |
| GSM131564 GSM131565 GSM131566 | ATGE_94_A ATGE_94_B ATGE_94_C | root |  |  |  |  |
|  |  |  |  |  |  |  |
| GSM131567 GSM131568 GSM131569 | ATGE_95_A ATGE_95_B ATGE_95_C | root |  |  |  |  |
|  |  |  |  |  |  |  |
| GSM131570 GSM131571 GSM131572 | ATGE_98_A ATGE_98_B ATGE_98_C | root |  |  |  |  |
|  |  |  |  |  |  |  |
| GSM131573 GSM131574 GSM131575 | ATGE_99_A ATGE_99_B ATGE_99_C | root |  |  |  |  |

| **Series:**  Flowers and pollen | | | | | | |
| --- | --- | --- | --- | --- | --- | --- |
| **Available at:**  http://affy.arabidopsis.info/narrays/experimentpage.pl?experimentid=152 | | | | | | |
|  |  |  |  |  |  |  |
| **GEO Access Identifier** | **Experiment Name** | **Tissue** |  | **GEO Access Identifier** | **Experiment Name** | **Tissue** |
|  |  |  |  |  |  |  |
| GSM131576 GSM131577 GSM131578 | ATGE_31_A2 ATGE_31_B2 ATGE_31_C2 | flowers stage 9 |  | GSM131609 GSM131610 GSM131611 | ATGE_43_A ATGE_43_B ATGE_43_C | flowers stage 15 stamen |
|  |  |  |  |  |  |  |
| GSM131579 GSM131580 GSM131581 | ATGE_32_A2 ATGE_32_B2 ATGE_32_C2 | flowers stage 10/11 |  | GSM131612 GSM131613 GSM131614 | ATGE_45_A ATGE_45_B ATGE_45_C | flowers stage 15 carpels |
|  |  |  |  |  |  |  |
| GSM131582 GSM131583 GSM131584 | ATGE_33_A ATGE_33_B ATGE_33_C | flowers stage 12 |  | GSM131615 GSM131616 GSM131617 | ATGE_53_A ATGE_53_B ATGE_53_C | flower stage 12 equivalent |
|  |  |  |  |  |  |  |
| GSM131585 GSM131586 GSM131587 | ATGE_34_A ATGE_34_B ATGE_34_C | flowers stage 12 sepals |  | GSM131618 GSM131619 GSM131620 | ATGE_54_A ATGE_54_B ATGE_54_C | flower stage 12 equivalent |
|  |  |  |  |  |  |  |
| GSM131588 GSM131589 GSM131590 | ATGE_35_A ATGE_35_B ATGE_35_C | flowers stage 12 petals |  | GSM131621 GSM131622 GSM131623 | ATGE_55_A ATGE_55_B ATGE_55_C | flower stage 12 equivalent |
|  |  |  |  |  |  |  |
| GSM131591 GSM131592 GSM131593 | ATGE_36_A ATGE_36_B ATGE_36_C | flowers stage 12 stamens |  | GSM131624 GSM131625 GSM131626 | ATGE_56_A ATGE_56_B ATGE_56_C | flower stage 12 equivalent |
|  |  |  |  |  |  |  |
| GSM131594 GSM131595 GSM131596 | ATGE_37_A ATGE_37_B ATGE_37_C | flowers stage 12 carpels |  | GSM131627 GSM131628 GSM131629 | ATGE_57_A ATGE_57_B ATGE_57_C | flower stage 12 equivalent |
|  |  |  |  |  |  |  |
| GSM131597 GSM131598 GSM131599 | ATGE_39_A ATGE_39_B ATGE_39_C | flowers stage 15 |  | GSM131630 GSM131631 GSM131632 | ATGE_58_A ATGE_58_B ATGE_58_C | flower stage 12 equivalent |
|  |  |  |  |  |  |  |
| GSM131600 GSM131601 GSM131602 | ATGE_40_A ATGE_40_B ATGE_40_C | flowers stage 15 pedicels |  | GSM131633 GSM131634 GSM131635 | ATGE_59_A ATGE_59_B ATGE_59_C | flower stage 12 equivalent |
|  |  |  |  |  |  |  |
| GSM131603 GSM131604 GSM131605 | ATGE_41_A ATGE_41_B ATGE_41_C | flowers stage 15 sepals |  | GSM131636 GSM131637 GSM131638 | ATGE_73_A ATGE_73_B ATGE_73_C | mature pollen |
|  |  |  |  |  |  |  |
| GSM131606 GSM131607 GSM131608 | ATGE_42_A ATGE_42_B ATGE_42_C | flowers stage 15 petals |  | GSM131639 GSM131640 GSM131641 | ATGE_92_A ATGE_92_B ATGE_92_C | flower |

| **Series:** Shoots and Stems | | | | | | |
| --- | --- | --- | --- | --- | --- | --- |
| **Available at:**  http://affy.arabidopsis.info/narrays/experimentpage.pl?experimentid=153 | | | | | | |
|  |  |  |  |  |  |  |
| **GEO Access Identifier** | **Experiment Name** | **Tissue** |  | **GEO Access Identifier** | **Experiment Name** | **Tissue** |
|  |  |  |  |  |  |  |
| GSM131643. GSM131644 GSM131645 | ATGE_2_A ATGE_2_B ATGE_2_C | hypocotyl |  | GSM131673 GSM131674 GSM131675 | ATGE_49_A ATGE_49_B ATGE_49_C | shoot apex, inflorescence |
|  |  |  |  |  |  |  |
| GSM131646. GSM131647 GSM131648 | ATGE_4_A ATGE_4_B ATGE_4_C | shoot apex, vegetative + young leaves |  | GSM131676 GSM131677 GSM131678 | ATGE_50_A ATGE_50_B ATGE_50_C | shoot apex, inflorescence |
|  |  |  |  |  |  |  |
| GSM131649. GSM131650 GSM131651 | ATGE_6_A ATGE_6_B ATGE_6_C | shoot apex, vegetative |  | GSM131679 GSM131680 GSM131681 | ATGE_51_A ATGE_51_B ATGE_51_C | shoot apex, inflorescence |
|  |  |  |  |  |  |  |
| GSM131652. GSM131653 GSM131654 | ATGE_8_A ATGE_8_B ATGE_8_C | shoot apex, transition |  | GSM131682 GSM131683 GSM131684 | ATGE_52_A ATGE_52_B ATGE_52_C | shoot apex, inflorescence |
|  |  |  |  |  |  |  |
| GSM131655. GSM131656 GSM131657 | ATGE_27_A2 ATGE_27_B2 ATGE_27_C2 | stem, 2nd internode |  | GSM131480 GSM131481 GSM131482 | ATGE_24_A ATGE_24_B ATGE_24_C | shoot apex, inflorescence |
|  |  |  |  |  |  |  |
| GSM131658. GSM131659 GSM131660 | ATGE_28_A2 ATGE_28_B2 ATGE_28_C2 | 1st node |  |  |  |  |
|  |  |  |  |  |  |  |
| GSM131661. GSM131662 GSM131663 | ATGE_29_A2 ATGE_29_B2 ATGE_29_C2 | shoot apex, inflorescence |  |  |  |  |
|  |  |  |  |  |  |  |
| GSM131664. GSM131665 GSM131666 | ATGE_46_A ATGE_46_B ATGE_46_C | shoot apex, inflorescence |  |  |  |  |
|  |  |  |  |  |  |  |
| GSM131667 GSM131668 GSM131669 | ATGE_47_A ATGE_47_B ATGE_47_C | shoot apex, inflorescence |  |  |  |  |
|  |  |  |  |  |  |  |
| GSM131670 GSM131671 GSM131672 | ATGE_48_A ATGE_48_B ATGE_48_C | shoot apex, inflorescence |  |  |  |  |

| **Series:**  Siliques and Seeds | | | | | | |
| --- | --- | --- | --- | --- | --- | --- |
| **Available at:**  http://affy.arabidopsis.info/narrays/experimentpage.pl?experimentid=154 | | | | | | |
|  |  |  |  |  |  |  |
| **GEO Access Identifier** | **Experiment Name** | **Tissue** |  |  |  |  |
|  |  |  |  |  |  |  |
| GSM131685 GSM131686 GSM131687 | ATGE_76_A ATGE_76_B ATGE_76_C | siliques, w/ seeds stage 3 |  |  |  |  |
|  |  |  |  |  |  |  |
| GSM131688 GSM131689 GSM131690 | ATGE_77_A ATGE_77_B ATGE_77_C | siliques, w/ seeds stage 4 |  |  |  |  |
|  |  |  |  |  |  |  |
| GSM131691 GSM131692 GSM131693 | ATGE_78_A ATGE_78_B ATGE_78_C | siliques, w/ seeds stage 5 |  |  |  |  |
|  |  |  |  |  |  |  |
| GSM131694 GSM131695 GSM131696 | ATGE_79_A ATGE_79_B ATGE_79_C | seeds, stage 6, w/o siliques |  |  |  |  |
|  |  |  |  |  |  |  |
| GSM131697 GSM131698 GSM131699 | ATGE_81_A ATGE_81_B ATGE_81_C | seeds, stage 7, w/o siliques |  |  |  |  |
|  |  |  |  |  |  |  |
| GSM131700 GSM131701 GSM131702 | ATGE_82_A ATGE_82_B ATGE_82_C | seeds, stage 8, w/o siliques |  |  |  |  |
|  |  |  |  |  |  |  |
| GSM131703 GSM131704 GSM131705 | ATGE_83_A ATGE_83_B ATGE_83_C | seeds, stage 9, w/o siliques |  |  |  |  |
|  |  |  |  |  |  |  |
| GSM131706 GSM131707 GSM131708 | ATGE_84_A ATGE_84_B ATGE_84_C | seeds, stage 10, w/o siliques |  |  |  |  |
