# Supplementary material for: Mutational Biases and Selective Forces Shaping the Structure of Arabidopsis Genes
Source: PLoS One. 2009 Jul 27;4(7):e6356. doi: 10.1371/journal.pone.0006356 (PMC2712092; doi:10.1371/journal.pone.0006356)
Supplement: Table S2 — List of MPSS experiments considered in our paper (0.03 MB DOC) [file pone.0006356.s002.doc]

**Table S2:** List of the MPSS experiments considered

| **MPSS Access Identifier** | **Tissue** | **Experiment Description** | **http** |
| --- | --- | --- | --- |
|  |  |  |  |
| INF | Influorescence | Infloresence - mixed stage, immature buds, classic MPSS | http://mpss.udel.edu/at/Library.php?lib=2&tag_length=17 |
| LEF | Leaves | Leaves - 21 day, untreated, classic MPSS | http://mpss.udel.edu/at/Library.php?lib=3&tag_length=17 |
| ROF | Root | Root - 21 day, untreated, classic MPSS | http://mpss.udel.edu/at/Library.php?lib=4&tag_length=17 |
| SIF | Silique | 24 to 48 hr post-fertilization, classic MPSS | http://mpss.udel.edu/at/Library.php?lib=5&tag_length=17 |
| GSE | Seedlings | Germinating seedlings | http://mpss.udel.edu/at/Library.php?lib=15&tag_length=17 |
