# Supplementary material for: Mutational Biases and Selective Forces Shaping the Structure of Arabidopsis Genes
Source: PLoS One. 2009 Jul 27;4(7):e6356. doi: 10.1371/journal.pone.0006356 (PMC2712092; doi:10.1371/journal.pone.0006356)
Supplement: Table S3 — Methods used in order to estimate the gene expression levels. (0.03 MB DOC) [file pone.0006356.s003.doc]

**Table S3:** Methods used in order to estimate the gene expression levels.

| **Method** | **Descriprion** |
| --- | --- |
|  | |
| DS-A | The Exeprimental unit is rapresented by the developmental stage. The expression level for each gene is obtained by averaging the expression values of all the experimental units. |
| DS-I | The Experimental unit is rapresented by the developmental stage. The expression level for each gene is obtained by averaging the expression values of only the experimental units in which the gene is actually expressed |
| DS-pE | The Experimental unit is rapresented by the developmental stage.The expression level for each gene is equal to the peak of expression taking in to account all experimental units. |
| O-A | The Experimental unit is rapresented by the organ. The expression level for each gene is obtained by averaging the expression values of all the experimental units. |
| O-I | The Experimental unit is rapresented by the organ. The expression level for each gene is obtained by averaging the expression values of only the experimental units in which the gene is actually expressed |
| O-pE | The Experimental unit is rapresented by the organ. The expression level for each gene is equal the peak of expression taking in to account all experimental units. |
